# Enhancing patient-clinician collaboration during treatment decision-making: study protocol for a community-engaged, mixed method hybrid type 1 trial of collaborative decision skills training (CDST) for veterans with psychosis

**DOI:** 10.1186/s13063-024-08127-4

**Published:** 2024-06-06

**Authors:** Emily B. H. Treichler, Lauren E. McBride, Elissa Gomez, Joanna Jain, Sydney Seaton, Kasey E. Yu, David Oakes, Dimitri Perivoliotis, Vanessa Girard, Samantha Reznik, Michelle P. Salyers, Michael L. Thomas, William D. Spaulding, Eric L. Granholm, Borsika A. Rabin, Gregory A. Light

**Affiliations:** 1 VA Desert Pacific Mental Illness Research, Education, and Clinical Center (MIRECC), San Diego, CA USA; 2 Department of Psychiatry, University of California San Diego, 9500 Gilman Drive 0804, La Jolla, CA 92093 USA; 3 UC San Diego Dissemination and Implementation Science Center, University of California San Diego, La Jolla, CA USA; 4 Herbert Wertheim School of Public Health and Human Longevity Science, University of California San Diego, La Jolla, CA USA; 5 VA San Diego Psychology Service, San Diego, CA USA; 6 University of Texas at Austin, Texas Institute for Excellence in Mental Health, Austin, USA; 7 Department of Psychology, Indiana University-Purdue University at Indianapolis, Indianapolis, IN USA; 8 Department of Psychology, Colorado State University, Fort Collins, CO USA; 9 Department of Psychology, University of Nebraska-Lincoln, Lincoln, NE USA; 10 Center of Excellence in Stress and Mental Health, San Diego VA, La Jolla, CA USA

**Keywords:** Recovery, Person-centered care, Implementation science, Schizophrenia, Shared decision-making

## Abstract

**Background:**

Patient participation in treatment decision making is a pillar of recovery-oriented care and is associated with improvements in empowerment and well-being. Although demand for increased involvement in treatment decision-making is high among veterans with serious mental illness, rates of involvement are low. Collaborative decision skills training (CDST) is a recovery-oriented, skills-based intervention designed to support meaningful patient participation in treatment decision making. An open trial among veterans with psychosis supported CDST’s feasibility and demonstrated preliminary indications of effectiveness. A randomized control trial (RCT) is needed to test CDST’s effectiveness in comparison with an active control and further evaluate implementation feasibility.

**Methods:**

The planned RCT is a hybrid type 1 trial, which will use mixed methods to systematically evaluate the effectiveness and implementation feasibility of CDST among veterans participating in a VA Psychosocial Rehabilitation and Recovery Center (PRRC) in Southern California. The first aim is to assess the effectiveness of CDST in comparison with the active control via the primary outcome, collaborative decision-making behavior during usual care appointments between veterans and their VA mental health clinicians, and secondary outcomes (i.e., treatment engagement, satisfaction, and outcome). The second aim is to characterize the implementation feasibility of CDST within the VA PRRC using the Practical Robust Implementation and Sustainability Model framework, including barriers and facilitators within the PRRC context to support future implementation.

**Discussion:**

If CDST is found to be effective and feasible, implementation determinants gathered throughout the study can be used to ensure sustained and successful implementation at this PRRC and other PRRCs and similar settings nationally.

**Trial registration:**

ClinicalTrials.gov NCT04324944. Registered on March 27, 2020. Trial registration data can be found in Appendix 1.

**Supplementary Information:**

The online version contains supplementary material available at 10.1186/s13063-024-08127-4.

## Background

The United States Department of Veterans Affairs (VA) significantly invests in veteran-centered care for all veterans and recovery-oriented care specifically for veterans with serious mental illness (SMI) [1]. Recovery-oriented care is built upon the recovery movement, originally championed by people with lived experience of SMI [2]. It prioritizes a holistic, strength-based approach to mental health care including personally identified goals that support increased joy, empowerment, and personal meaning regardless of whether symptoms improve [3]. The VA grounded its nationally implemented Psychosocial Rehabilitation and Recovery Center (PRRC) programs around this model, including affiliated training programs for mental health clinicians [4].

Patient participation in treatment decision-making is a key element of recovery-oriented care [5]. Meaningful patient participation facilitates treatment personalization and fosters an empowering treatment process. Further, involvement in mental health treatment decision-making is preferred by 85% of veterans with SMI [6] and is associated with desirable outcomes, including improved treatment engagement, empowerment, and sense of well-being [7–9]. However, rates of veteran involvement in decision-making remain low in VA settings [8], indicating an important gap in meeting the promise of recovery-oriented care in the VA.

Shared decision-making (SDM) is a commonly used approach to increase patient participation in the decision-making process. However, there are significant barriers to its effectiveness and implementation feasibility in usual care settings, especially for people with SMI [10]. For example, most decision aids tend to be focused on a specific decision (e.g., which medication to take) based on clinical guidelines, limiting their utility for complex and dynamic treatment contexts with many decisions, including decisions without clear best practices (e.g., which supported housing program to choose; whether to apply for disability) [11]. Collaborative decision-making (CDM) [10] was developed to respond to these barriers. CDM emphasizes patient power and participation across all levels of decision-making and prioritizes patient values, needs, preferences, and cultural context in decisions, ensuring its alignment with the recovery model. CDM restructures the decision-by-decision approach of most decision-making models to a collaborative, ongoing approach, given that the chronic concerns that people with SMI often face typically require treatment plans and decisions to be iteratively recalibrated.

Collaborative decision skills training (CDST) is a novel intervention to increase CDM among veterans with SMI. CDST is an empowerment-oriented group intervention that was developed for civilians with SMI to increase comfort, confidence, knowledge, and skills associated with treatment decision-making [12]. The initial civilian pilot was promising, indicating that CDST was feasible and showed initial effectiveness among 21 people with SMI participating in a day rehabilitation program [11]. CDST has since been adapted for VA PRRCs [13] and has undergone a small (*N* = 9) one-armed open trial to assess feasibility and initial effectiveness among veterans with psychosis participating in VA PRRC services at a VA in Southern California [10, 14]. Feasibility data was strong, and preliminary outcome data showed that veterans who participated in CDST were more involved in treatment decision-making and more active in treatment appointments overall at post-intervention and 3-month follow-up evidenced by both observational and quantitative data. Secondary outcomes including personal recovery, empowerment, and treatment engagement also improved. However, a larger study is needed to confirm CDST as a viable candidate to improve CDM among veterans participating in VA PRRCs. Therefore, in this protocol, we describe a hybrid type 1 randomized control trial (RCT) that will assess CDST’s effectiveness as compared to an active control (AC) in the PRRC context and further evaluate the feasibility of implementing CDST into routine care at PRRCs.

### Study rationale and aims

Given this promising but preliminary evidence that CDST may be a feasible and effective approach to increase CDM among veterans with SMI in VA PRRCs, an appropriate next step is a hybrid type 1 superiority trial to systemically evaluate the effectiveness and implementation feasibility [15, 16]. The hybrid study approach facilitates efficient movement in the research-to-practice pipeline and ensures that effectiveness results are accurately contextualized by implementation data. Hybrid type 1 studies prioritize testing the effectiveness of an intervention while capturing information about the intervention’s implementation potential [15]. Using the guiding questions from Curran et al. [16], our rationales for choosing a hybrid type 1 design are as follows: (1) stronger effectiveness data for CDST is still needed, (2) we will adapt the intervention for this setting throughout the trial, and (3) we are at the stage of examining the implementation determinants rather than testing an implementation strategy.

**Fig. 1:**
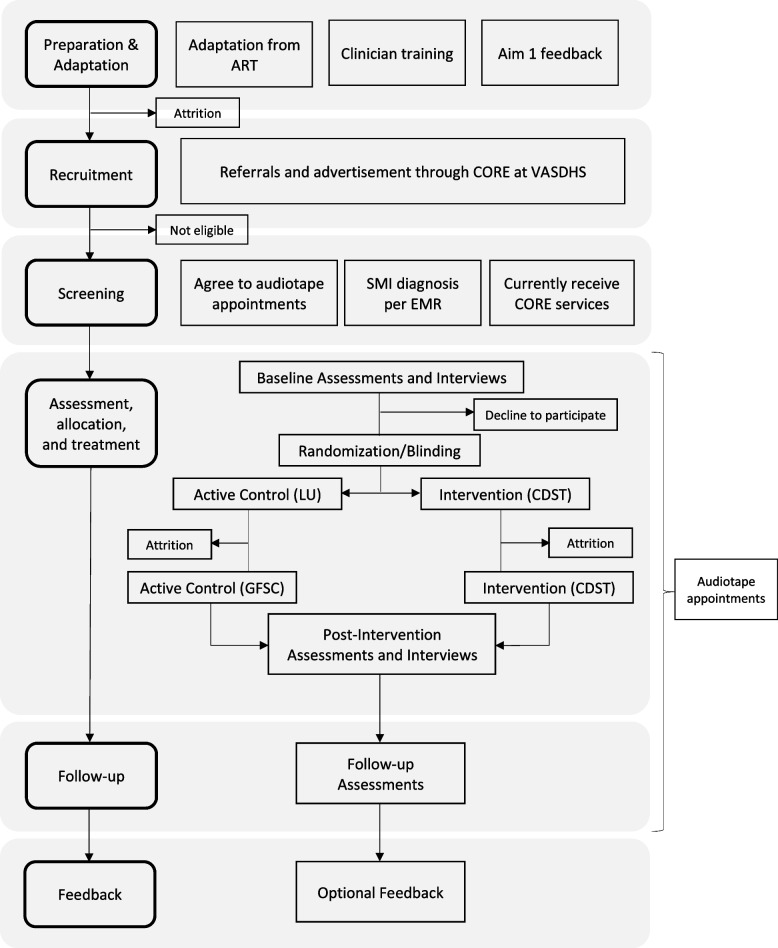
CONSORT diagram

We will use a mixed-methods approach guided by the Practical Robust Implementation and Sustainability Model (PRISM) to systematically evaluate CDST’s effectiveness and implementation feasibility [17, 18]. PRISM extends the widely utilized Reach, Effectiveness, Adoption, Implementation, and Maintenance (RE-AIM) framework to collect information on multilevel contextual factors that may impact implementation outcomes [19]. PRISM has successfully been used in the past within the VA healthcare system to guide program evaluation and implementation [20, 21]. A CONSORT diagram for this study can be found in Fig. 1 [22].

The primary aim of this study will be to assess the effectiveness of CDST among veterans with psychosis participating in a VA PRRC compared to the active control group using a community-engaged, mixed methods approach. Within this aim, we hypothesize that CDST will demonstrate effectiveness via the primary outcome, collaborative decision-making behavior during usual care appointments between veterans and their VA mental health clinicians. We further hypothesize that veterans in CDST will show greater improvements than veterans in the active control in the secondary outcomes: treatment engagement, treatment satisfaction, and treatment outcomes (e.g., empowerment, symptom severity, social skills, and goal attainment). Additionally, we will explore the impact of CDST on acute service use and clinician engagement in collaboration. The secondary aim of this study is to characterize the implementation feasibility of CDST within VA PRRCs using the PRISM/RE-AIM framework [17]. Within this aim, we hypothesize that CDST will be feasible to implement, as indicated by veteran satisfaction; CDST attendance, engagement, and at-home practice completion; and therapist fidelity. We will also describe the other major components of RE-AIM (i.e., reach, adoption, and maintenance) but make no hypotheses given that this is a hybrid type 1 trial. We will use qualitative methods to identify implementation determinants (i.e., PRISM contextual domains) in the PRRC context to inform future implementation of CDST.

## Methods

This study used the SPIRIT reporting guidelines for clinical trial protocols [23]. This protocol is aligned with the 2013 SPIRIT Checklist for *Trials*, and a completed checklist can be found in attachment 1.

### Community engagement approach

This study will engage key community partners including veterans with lived experience of SMI, VA clinicians, and VA administrators in multiple ways. First, community partners provided input during the original conception of this study and then contributed to adaptations to the protocol following the open trial. Second, as described in the “Adaptation resource team and adaptation schedule” section, veterans and clinicians comprise an ongoing team contributing to adaptations to CDST since the pre-implementation phase and throughout the entirety of this study. Third, veterans and clinicians may provide qualitative feedback following their participation if they choose. Fourth, a VA administrator serves as a senior investigator on this project. Fifth, veterans and clinicians who participate in the adaptation resource team and clinicians who provide the intervention have the opportunity to participate in research and dissemination activities (e.g., this paper includes one veteran co-author, three clinician co-authors, and one administrator co-author).

### Setting

Veterans currently participating at a PRRC in Southern California will be recruited for this study. Nationwide, PRRCs provide recovery-oriented outpatient services including individual therapy, group therapy, medication management, peer support, and vocational rehabilitation to veterans with SMI [24]. VHA Directive 1163 (2019) defines SMI as a mental, behavioral, or emotional disorder that meets three criteria: (1) a single episode with unremitting symptoms or recurring symptom episodes that result in (2) impairments that substantially impact daily living and (3) Global Assessment of Functioning (GAF) score of 50 or below, indicating significant functional impairments [25]. Although the primary diagnostic emphasis for PRRCs is on veterans with severe manifestations of psychosis, many also provide care to veterans with severe PTSD and mood disorders without psychotic features. The target PRRC in this study is distinct in that it serves exclusively veterans with primary, non-substance-induced psychotic disorders. As of 2023, two-thirds of the local PRRC veteran population identify as male, and the racial/ethnic breakdown of veterans served at the clinic is 44% White, 22% Hispanic or Latina/o/x, 17% Black, 11% Asian, and 6% Native American [26].

### Sample size

We conducted a priori power analysis using G*Power 2 based on repeated measures ANOVA, which will yield consistent results with the proposed linear-mixed models, if the assumptions of ANOVA are not violated [27]. We used 80% power and an alpha = 0.05 in our calculations, which are widely accepted parameters for power calculations in clinical trials [28]. Based on this, we plan to recruit 72 veterans over the 3-year study period for a final sample of 58 veterans (estimating ~ 20% attrition based on RCTs for similar interventions with SMI population) [29]. With 58 participants and 3 time points (baseline, post-intervention, and 3-month follow-up), we will have 80% power to detect small-to-medium effect sizes (*f* = 0.17) with alpha = 0.05 (two tail) and an expected correlation between repeated measures of 0.5.

### Participant recruitment

**Fig. 2:**
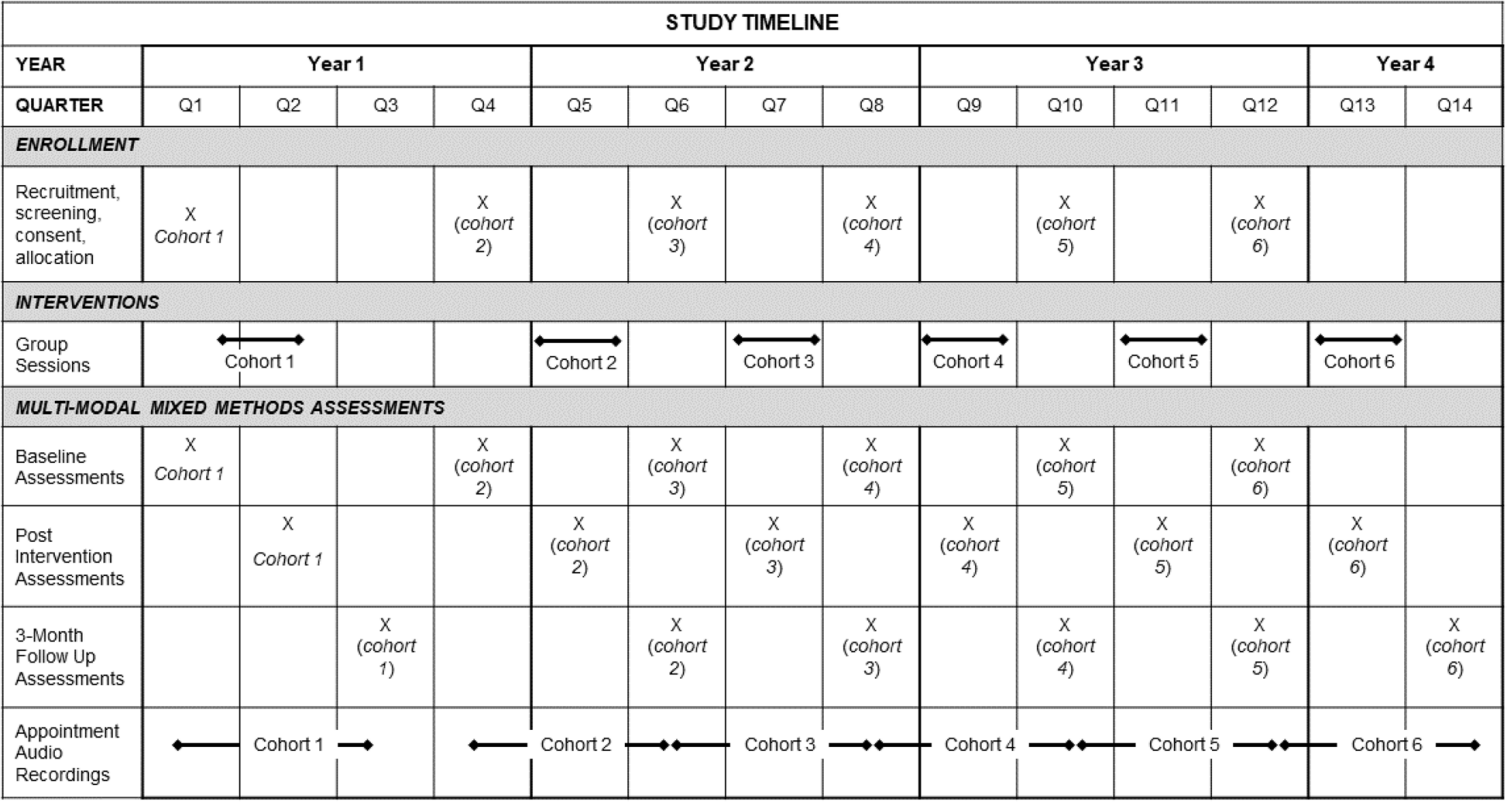
Study timeline

We will recruit participants in six cohorts over the course of the 3-year study period (2 cohorts per year) to ensure that we can iteratively adapt CDST and that we can reach our recruitment goal (see Fig. 2). The inclusion criteria for the RCT are as follows: (1) receive services at the PRRC at the time of recruitment; (2) have an SMI diagnosis documented in the electronic medical record, which includes schizophrenia, schizoaffective disorder, delusional disorder, an unspecified non-substance induced psychotic disorder, or a major affective disorder with psychotic features; (3) are age 18 or above; (4) are fluent and literate in English; and (5) agree to have a subset of their mental health treatment appointments audio-recorded. The exclusion criteria for the RCT are as follows: (1) have a primary substance use or organic neurological disorder diagnosis and (2) determined by PRRC staff/study staff to be at significant risk of symptom exacerbation or risk of violence too high to manage in the study setting. There are no restrictions on the use of medications or other therapies for study participants in the best interest of participant well-being and to align with our overall hybrid 1 effectiveness-implementation approach, i.e., to align as much as possible with usual care processes. In keeping with current clinical practices at the PRRC, participants will be recruited via referrals from PRRC clinicians and flyering in the clinic waiting area. Additionally, as research has shown that handwritten letters are an effective study recruitment strategy for the veteran population [29], handwritten letters and flyers will be sent to veterans on the PRRC census.

### Adaptation resource team and adaptation schedule

Adapting manualized interventions for specific treatment contexts is critical to ensure optimal fit, and as a result, sustained adoption and implementation [30]. Although CDST has been delivered in the VA population, we will continue to adapt the intervention to increase relevance and fit for the population and clinical context. These adaptations will change CDST’s *form* without changing its core *functions*, as described by Pérez Jolles [31]. Specifically, while specific activities or other aspects of the intervention (e.g., the appearance or wording of the manual) may change, none of the components believed to be “essential ingredients” (functions) to CDST’s effectiveness will be removed or fundamentally altered. This allows for consistent monitoring of fidelity based on these functions. We will use an abbreviated version of the Intervention Mapping (IM Adapt) [32] process to adapt CDST and its training and delivery materials, in concordance with the process used in the pre-implementation adaptation of CDST [10].

The adaptation resource team (ART) includes veterans with SMI, VA clinicians, and members of the research team originating from the pre-implementation adaptation and open trial of CDST [10]. After each cohort, the CDST clinician and veterans who participated in CDST will be invited to join the ART. Following each trial cohort, the ART will meet twice to discuss potential adaptations to CDST based on feedback from trial participants. These meetings use a collaborative, iterative process to identify and implement feasible, fidelity-consistent adaptations that meet both veteran and clinician needs. We will alternate between longer and shorter adaptation periods to balance between pragmatism and rigor. During longer adaptation periods, we will complete a rapid matrix analysis of the suggestions made during trial participant qualitative interviews to identify possible adaptations to present to the ART [33]. Shorter adaptations will rely only on field notes created during these qualitative interviews to reduce turnaround time [34]. In addition to ART meetings, there will be periodic reflection meetings throughout the RCT to ensure documentation of implementation phenomena, such as unplanned modifications, challenges, and adaptations related to clinical delivery and data collection [34].

Adaptations and modifications will be systematically documented using the RE-AIM enhanced, expanded framework to reporting adaptations and modifications to evidence-based interventions (FRAME) [10, 31, 35]. This expanded model is intended to capture suggestions and their context and rationale [10, 30]. We additionally included items to measure the size and scope of the adaptation or modification [36], whether the adaptation was implemented (and if not, why not), and any specific population the adaptation was targeted towards. FRAME may contribute to a successful and efficient implementation of CDST in other healthcare settings or populations by supporting the identification of relevant and feasible adaptations.

### Intervention and control groups

Veterans will be randomly assigned to participate in either the experimental group (CDST) or the active control group (Leveling Up). Both groups consist of ten 1-h sessions. CDST has six core functions [31]:

1. Empowerment-focused therapeutic approach that enhances overall and specific feelings of empowerment related to recovery and participation in mental health care
2. Skills training strategies to improve participants’ abilities to initiate and engage in collaborative decision-making
3. Training in specific skills relevant to collaborative decision-making, such as identification of treatment decisions, assertive communication, conflict resolution, and problem-solving
4. Psychoeducation on relevant topics, such as what collaborative decision-making is
5. Increasing comfort and confidence in participation in treatment decision-making processes
6. Supporting participants in problem-solving about barriers to collaborative decision-making

The active control (AC) for this study, Leveling Up, is adapted from goal-focused supportive contact (GFSC) [37]. Leveling Up provides a semi-structured, encouraging environment for veterans to share their experiences and receive support from the therapist and other veteran participants along with psychoeducation and resources without receiving exposure to any of the core functions of CDST. Each session will be composed of an initial check-in, an education module during which the clinician will provide information and resources on a separate topic each week, and time for discussion about relevant themes brought up in the session. An additional component, befriending, will be implemented at the clinician discretion and will involve casual conversation about neutral topics with the intent of building rapport. Topics covered in each session of CDST and Leveling Up can be found in Table 1.

**Table 1:** Topics covered in intervention and active control groups by week

Session number/week	Intervention	Active control
1	Intro to collaborative decision-making (CDM)	Positive symptoms
2	CDM and your treatment team	Negative symptoms
3	Setting treatment goals and making treatment decisions	Depression
4	Being assertive with your treatment team	Anxiety
5	Speaking up and dealing with conflict	Substance use
6	Introduction to problem-solving	Employment
7	Applying problem-solving to your mental health care	Personal relationships
8	CDM review: putting it all together activity	VA resources and benefits
9	Applying CDM to your mental health	Medication
10	CDM in your life and wrapping it up	Physical health

Both CDST and AC will be delivered by a master’s level or above, usual care clinician at the PRRC. Clinicians will be trained by the principal investigator to deliver both interventions. Additionally, the research team will complete live fidelity monitoring of four sessions of each intervention cohort using standard fidelity checklists [12]. Clinicians will complete a self-assessment of fidelity including any intentional or unintentional deviations from fidelity following each session [38].

### Randomization and blinding

Veterans will be randomized on a 1:1 ratio in blocks of four into CDST or AC following cohort baseline assessment completion. Given that the overall VA population is approximately 92% men [39], randomization will be stratified by gender to ensure a balanced sample*.* An allocation table generated in R will be implemented in the REDCap randomization module by a study staff member [40–42]. Throughout the study, we will monitor the demographics of veterans by group, such as race/ethnicity, symptom severity, and age, and incorporate additional stratification if the groups are unbalanced with guidance from a statistician.

The research staff will be pragmatically divided into blinded and unblinded groups to ensure that veteran group assignment does not bias outcome assessment and analysis. Only the study staff who are blinded will conduct key outcome assessment activities (i.e., deliver quantitative assessments and code audio-recorded treatment appointments), and they will be restricted from completing activities that could unblind them (e.g., reviewing health records). We will monitor blindness throughout the study by surveying blinded study staff on the group assignment of each veteran. If a blinded study staff member correctly guesses the group assignments of 75% of the veterans, they will be moved to the unblinded group for that cohort. This approach is considered a low risk of bias by the Cochrane Risk of Bias Tool for randomized trials.

### Assessments

Veterans will complete a quantitative assessment and a qualitative interview at three time points: baseline, post-intervention, and 3-month follow-up (see Fig. 2). Participants randomized into the CDST group will have the option to complete an additional qualitative interview post-intervention to collect feedback on CDST for future adaptations. All veterans will be compensated for $25 per qualitative interview and $40 per quantitative assessment. Veterans will not be compensated for their participation in intervention-related activities to minimize the impact on associated outcomes. CDST clinicians will complete a qualitative interview at baseline and post-intervention. Additionally, usual care clinicians in the PRRC working with each veteran will complete brief surveys (e.g., on service engagement) in the study at baseline, halfway through the intervention, post-intervention, and 3 months following the intervention.

### Participant study engagement and retention

We will employ a variety of strategies to engage and retain study participants. These include scheduling study appointments at times most convenient for participants, offering both in-person and virtual options for study appointments, allowing participants to take breaks during assessments or split assessments into multiple days, and giving participants the option to skip questions during assessments. Participants who withdraw from CDST or AC, along with those who leave PRRC care for any reason, will still have the option to complete assessments.

### Measures

#### Primary outcome

The primary outcome is veteran-provider collaboration in the usual care setting. We define collaboration as the presence of specific behaviors, including asking questions, stating preferences, expressing agreement or disagreement, and making requests. We will record up to eight mental health treatment appointments for each participant throughout the study. We will use two validated coding systems to measure collaboration in these audio-recorded usual care appointments: the Shared Decision-Making Coding System (SDM-CS) and the Consumer-Created Opportunities for Active Involvement Coding System (CCOAI-CS). The SDM-CS measures patient-provider collaboration during treatment decision-making [8]. The CCOAI-CS measures consumer-initiated (meaning veteran, in this context) collaborative behaviors throughout the appointment regardless of whether a decision occurs [43]. Both coding systems were piloted in the study setting and adapted by the developer. Blinded research assistants will be trained in the coding systems and will need to code two consecutive appointments with 70% agreement or greater with a trained research assistant before primary coding alone. Research assistants will double code and meet for consensus for 80% of appointments during cohort 1, 70% of appointments during cohort 2, and 50% of appointments in cohorts 3, 4, 5, and 6. Although this method was feasible during the open trial of CDST, there were barriers to recording as many appointments as desired, such as variability in appointment frequency. We have adapted our data collection strategy to optimize feasibility for the RCT: we will record appointments throughout the study instead of at fixed time points, allow for two recordings with the same clinician at one time point (e.g., at baseline), and record any VA mental health appointments given that many veterans at this PRRC work with non-PRRC VA psychiatrists or individual therapists. While we will use both the SDM-CS and CCOAI-CS to support a comprehensive assessment of collaborative behavior in both decisional and non-decisional contexts, the primary outcome will be assessed using the SDM-CS (decisional contexts) by assessing changes over time from baseline to three month follow-up using the linear mixed model described below.

### Secondary outcomes

#### Secondary outcome 1: Collaborative decision-making perceptions and preferences

Veteran’s perceptions and preferences will be measured using the Shared Decision-Making Scale for Mental Health (SDM-MH-9), Collaborative Decision-Making Approach Measure (CD-MAM), and Problem-Solving Decision-Making Questionnaire for Mental Health (PSDMQ-MH), which were piloted in the open trial [12, 44, 45]. Following the open trial, we added the Perceived Involvement in Care Scale (PICS) [46] to collect quantitative data on each veteran’s perceptions about their own decision-making behavior.

#### Secondary outcome 2: Treatment engagement and motivation

Treatment engagement and motivation will be measured using the following: veterans’ non-study related appointment attendance at the PRRC; the Situational Motivational Scale for Schizophrenia Research [47], a 16-item self-report measure; and the Service Engagement Scale (SES) [48], a 14-item clinician-report measure. All these measures were piloted in the open trial. We removed a brief survey included in the open trial about veterans’ interest and engagement in specific rehabilitative services offered at the PRRC because the specific services offered change approximately every quarter, resulting in challenges with collecting and interpreting data. Instead, we will collect data on whether a veteran is still enrolled in the PRRC at the post- and follow-up time points, as well as the reason for withdrawal from the program (if applicable).

#### Secondary outcome 3: Treatment satisfaction

Treatment satisfaction will be measured using the Client Satisfaction Questionnaire (CSQ) [49]. No changes were made from the open trial.

#### Secondary outcome 4: Improved treatment outcomes

Treatment outcomes will be measured using the Canadian Occupational Performance Measure (COPM) [50], Maryland Assessment of Recovery in Serious Mental Illness (MARS) [51], Social Skills Performance Assessment (SSPA) [52], Goal Attainment Scaling (GAS) [53], and Personal and Social Performance Scale (PSP) [54], all of which were used in the open trial. Following the open trial, a minority of usual care clinicians requested a briefer clinician survey to reduce the burden. We therefore removed the gold standard symptom severity measure, the Brief Psychiatric Rating Scale (BPRS) [55]. We replaced it with the Clinical Global Impressions Scale, which is a validated, two-item measure [56], and added the BASIS-24 [57], a self-report symptom severity measure. Given that this intervention is not primarily targeting symptoms, these measures, which also have established validity if less sensitivity [58, 59], are appropriate. Additionally, in the open trial, we included the Empowerment Scale [60] as an exploratory outcome. In this RCT, we are including it as a secondary outcome measure given the importance of empowerment to personal recovery and CDM.

### Exploratory outcomes

#### Exploratory outcome 1: Clinician attitudes and behavior

We will measure changes in clinician behavior during treatment decision-making using the SDM-CS, which is described above. Additionally, we will use qualitative interviews to assess clinician attitudes related to treatment decision-making.

#### Exploratory outcome 2: Acute service use

We will collect data on emergency room visits, crisis calls, and inpatient hospital stays from participant medical records 12 months prior to the start of the study through 3 months following intervention completion.

### Implementation outcomes

Our primary implementation outcome is implementation feasibility using satisfaction, participation, and fidelity as metrics. Quantitative measures will include patient-reported satisfaction [12]; clinician-reported attendance, engagement, and at-home practice completion; and expert-rated fidelity as described in “fidelity monitoring.”

Secondary implementation outcomes will aim to characterize the other components of RE-AIM: Reach, Adopt, and Maintenance. Reach will be assessed by characterizing veterans who are screened, enrolled in the study, and completed CDST compared to veterans: (1) in the PRRC as a whole, (2) who are screened but do not enroll, (3) who enroll and are randomized to CDST but do not complete the intervention, and (4) AC completers. Adoption will be assessed by characterizing the PRRC and clinicians who choose to train and deliver CDST relative to the clinicians in the PRRC as a whole and to the AC condition. Maintenance will be assessed qualitatively given that this is a hybrid type 1 study and will capture veteran and clinician perspectives on whether CDST can and should be sustained in this setting, including sustainment determinants.

These data will be integrated with (1) veteran and clinician qualitative interviews including implementation barriers and facilitators organized by the PRISM/RE-AIM domains and (2) periodic reflections of implementation successes, challenges, and determinants. These data will allow us to characterize the implementation feasibility across the PRISM/RE-AIM model, including a comprehensive picture of determinants from multiple perspectives throughout the study.

### Qualitative interviews

Qualitative interviews will apply the localist approach to interviewing as a strategy to balance between our goal to remain as neutral as possible to avoid biasing interviewee responses while still needing to develop rapport and trust with interviewees and assess interviewee responses contextually [61–63]. We will therefore construct semi-structured interview guides which use open-ended questions so as not to lead questioning (e.g., asking about both benefits and harms) including both pre-set prompts and spontaneous follow-up questions to increase richness and ability to be responsive to each participant’s experience. Each participant will be interviewed by the same interviewer (the first author) at every time point to support trust development, with one or more secondary interviewer(s) with the participant’s permission. Interviewers will write field notes following each interview to provide key context, identify challenges, and support the development of themes and meta-inferences.

### Data analysis

#### Quantitative data quality assurance

The research staff will examine the quantitative data to identify unusual or invalid entries. If the source of the error cannot be identified and corrected (e.g., REDCap coding error), it will be converted to a missing data point. Outliers will be retained except in cases of credible invalidity (e.g., age entries lower than 18). We will assess whether data are missing completely at random (MCAR); if they are not, we will use multiple imputation or another appropriate method depending on the analysis to assure data quality.

#### Quantitative data analysis

We will use a linear mixed-effects model to complete an “intention-to-treat” analysis on all quantitative data, which is a gold standard approach for handling missing data points without having to remove a participant entirely from the dataset [64]. The outcome variable for each model will be the observed value at each time point. Models will include random intercepts and slopes for time, as well as fixed effects for group assignment, time, and the group-by-time interaction. We will include a secondary analysis to assess possible cohort effects including the impact of adaptations; this analysis will include all of the effects already mentioned with the addition of a fixed effect for the cohort. All statistical tests will use a two-tailed significance level of alpha = 0.05. We will also calculate Cohen’s *d* effect sizes for each measure by evaluating within-group change from baseline to post-intervention and from baseline to follow-up to understand the clinical significance of the results [65]. We will not perform a formal interim analysis.

#### Qualitative data analysis

Qualitative data will be composed of interview transcripts, field notes from interviews, and periodic reflections. Most data will be analyzed using a directed content analysis approach, meaning that codes will be developed a priori based on the PRISM/RE-AIM framework and theory-based interview guide [66, 67]. Additional codes will be added if they are identified by analysts. Baseline qualitative interviews with veterans will be used to identify their experiences seeking mental healthcare treatment, treatment engagement, how they make treatment decisions, and level of involvement. Interviews post-intervention and 3 months following their group will compare any changes made in treatment decision-making, patient-provider relationships, and feedback on either CDST or AC. Clinician interviews before and after facilitating the group will identify feasibility and future directions for CDST and Leveling Up in the context of the PRRC. The adaptation analyses will also rely on rapid analysis to support the ability to efficiently adapt between cohorts [33, 68]. During the short adaptation phases, field notes will be translated into template memos based on key themes, quotes, and specific adaptation-relevant suggestions made by participants. During the long adaptation phases, both qualitative interview transcripts and field notes will be used to create the template memos.

#### Mixed methods integration

The quantitative and qualitative data from this study will be integrated to support triangulation and deepen our understanding of the results [69]. Specifically, data will be integrated using a matrix approach [70] to allow for the interpretation of related data, including convergent and divergent results (e.g., the SDM-CS with survey and interview data about decision-making experiences). We will use a joint display analysis to support the integration of different data sources and types [71, 72]. Joint display analysis is a visual qualitative analytic approach to integrate quantitative and qualitative approach, typically by using displays to organize thematically related data (e.g., data about decision-making) to facilitate visual and contextual analysis and interpretation of associated data sources and achieve deeper understanding of complex data sets. Past work suggests that consistency in organizational and visual strategies, clarity about integration approaches, and use of theory to design the display is key to success [71, 72], so we will use these strategies for our displays and analysis. For example, this approach could facilitate the identification of key factors supporting improved CDM among veterans who benefit from CDST, while also identifying possible barriers for veterans who did not benefit (see Table 2 for a hypothetical example). Veteran and clinician partners will be highly engaged in the integration process to ensure relevance and meaningfulness to the target population.

**Table 2:** Example of joint display analysis

CDST benefits	Change in CDM^a^	Change in veteran-initiated collaborative behavior^b^	Beliefs about CDM at post-intervention	Strategies used to support CDM at follow-up
Yes (*n = XX*)	*X to X%*	*X to X%*	Quote from a participant who benefitted	Quote from a participant who benefitted
			Quote from a participant who benefitted	Quote from a participant who benefitted
No (*n = XX*)	*X to X%*	*X to X%*	Quote from a participant who did not benefit	Quote from a participant who did not benefit
			Quote from a participant who did not benefit	Quote from a participant who did not benefit

^a^Percent of treatment decisions recorded that were coded as “collaborative” or “veteran-led”

^b^CCOAI-CS translated to percent score

### Data management

All data for the study will be stored electronically and password-protected behind the secure VA firewall. Quantitative assessment data and chart review data will be collected and stored in the secure VA REDCap database [40, 41]. Each study participant will be assigned a unique, randomly generated ID code, and identifiable data will be stored separately from the data collected during the study. Access to identifiable data will be limited to approved research team staff on an as-needed basis.

### Data monitoring

Because this is a single-site study with no more than minimal risk to participants, there is no data safety monitoring board. However, the study staff will monitor participants’ well-being throughout the study, including exacerbation in psychiatric symptoms, and will report any adverse events or unexpected study side effects immediately to the IRB. The study staff will be embedded in the clinical context, including attending clinical team meetings, throughout the study period, to aid the ability to monitor participant well-being.

### Dissemination policy

The results from this trial will be published in professional journals and shared at relevant academic conferences and community partner meetings. We will only share de-identified datasets used in the analysis, and datasets will only be shared with researchers or other partners that provide a rationale and clear plan for the use of the data. The full study protocol will be made available on the ClinicalTrials.gov website. We will employ the criteria for authorship outlined by the International Committee of Medical Journal Editors [73]. In accordance with these criteria, the authors listed in this manuscript will be at least acknowledged in future publications related to this research.

## Discussion

Patient participation in treatment decision-making facilitates active exploration and integration of individual needs, preferences, and cultural context, enabling treatment to serve a purposeful role in the recovery process [74]. Current patient involvement models have distinct implementation barriers and, in practice, often stop short of true empowered decision-making. For example, clinicians still choose whether and how to implement these approaches, often meaning that clinicians only offer it to symptomatically stable patients or only include patients by providing psychoeducation about diagnosis or treatment rather than engaging patients in the decision-making process itself [11, 74]. To challenge these norms and meaningfully empower people with SMI, it is essential to uplift individuals as self-advocates and leaders in their own recovery [75]. For many, this requires gaining relevant knowledge, skills, comfort, confidence, and support to decide the extent and context in which they wish to engage in CDM [11, 76].

Therefore, this trial aims to understand whether CDST is an effective and feasible mechanism to support veterans with psychosis to increase collaboration during their usual care mental health treatment appointments. This study has significant strengths that will support our ability to achieve this goal. The first is our community-engaged approach, with a years-long relationship with the PRRC, including many of our veteran and clinician partners. This increases the research team’s understanding of the complex dynamics of a busy and constantly evolving VA PRRC, facilitating both clinical relevance of our intervention and design approach and allowing for pragmatic decision-making to support our ability to execute the study. For example, following the open trial, the main research team met with several clinician and veteran partners to review this study and adjust the protocol based on the successes and obstacles in the open trial (e.g., replacing the BPRS with the CGI and BASIS). Our continued relationship with clinical, operational, and veteran partners will facilitate our ability to execute this protocol successfully while being responsive to the needs of VA usual care, enhancing the utility of our findings for clinical delivery. The second strength is our complex mixed method design, which allows for the collection of ecologically valid, multifaceted observational data (the SDM-CS and CCOAI-CS), usual care EMR data, and gold standard quantitative and immersive qualitative data. This approach is both scientifically rigorous and clinically relevant and will support a deep understanding of CDST specifically and decision-making experiences of veterans in PRRCs broadly. Further, our thorough approach to documenting adaptations will support future evaluations and implementations of CDST in both similar and dissimilar contexts. Finally, information gleaned from the implementation aim will support the development of strategies to facilitate the future implementation of CDST.

### Expected challenges and limitations

A potential challenge of this study is the collection of audio recording data. As described above, we intended to audio record two treatment appointments per participant at each of the four time points during the open trial but experienced challenges including appointment cancelations and variability in how often veterans scheduled appointments. Additionally, although the PRRC offers medication management, all of the open trial participants worked with an outside prescriber. In this study, therefore, we extended audio recordings to include any VA mental health appointments and will record appointments throughout the duration of the study instead of at fixed time points to maximize our ability to capture these appointments. Additionally, although the goal is to capture eight appointments per participant, we are able to analyze the primary outcome data with fewer time points per participant.

There are limitations to this study’s methodology. As a hybrid effectiveness-implementation study, we will learn much about how CDST performs in a usual care treatment setting and maximize external validity; however, there are natural trade-offs in terms of internal validity. For example, as noted above, participants will not be restricted in terms of medications or other therapies. While we will randomize and monitor our groups for symptom severity and co-occurring disorders, it would be both infeasible and counter to our implementation aims to eliminate involvement in other aspects of a PRRC, a complex service environment. Additionally, there are practical limitations of our study given that this is a single-site clinical trial with a relatively small team including undergraduate research assistants; for example, we will not be able to maintain the same audio coders or quantitative assessors for the entire 3-year study period. Future research with additional sites and more resources will be able to assess the generalizability of this study’s findings across a greater number of VAs, a longer follow-up period, and allow for the assessment of moderators.

## Conclusion

This study will identify whether CDST is effective for veterans with psychosis and feasible for implementation in VA PRRCs. Regardless of the outcome, the complex mixed methods data will support the ongoing effort to realize the vision of recovery-oriented care in the VA.

## Trial status

Protocol version 8, July 26, 2023. Recruitment for this study began in August of 2022 and is expected to conclude in early 2025. Twenty veterans were enrolled in the study at the time that this protocol was submitted.

### Supplementary Information

**Supplementary Material 1.**

**Supplementary Material 2.**

**Supplementary Material 3.**
