# Supplementary material for: Enhancing patient-clinician collaboration during treatment decision-making: study protocol for a community-engaged, mixed method hybrid type 1 trial of collaborative decision skills training (CDST) for veterans with psychosis
Source: Trials. 2024 Jun 6;25:363. doi: 10.1186/s13063-024-08127-4 (PMC11155075; doi:10.1186/s13063-024-08127-4)
Supplement: Supplementary file 1 — Supplementary Material 1. [file 13063_2024_8127_MOESM1_ESM.docx]

Appendix 1. Trial Registration Data Set

| **Data type** | **Information** |
| --- | --- |
| Primary registry and trial identifying number | ClinicalTrials.gov NCT04324944 |
| Date of registration in primary registry | March 27, 2020 |
| Secondary identifying numbers | D3079-W |
| Source(s) of monetary or material support | VA Rehabilitation Research and Development Service |
| Primary sponsor | VA Rehabilitation Research and Development Service |
| Contact for public queries | Emily Treichler, PhD (Emily.Treichler@va.gov) |
| Contact for scientific queries | Emily Treichler, PhD (Emily.Treichler@va.gov) |
| Public title | Adapting and Examining Collaborative Decision Skills Training Among Veterans With Serious Mental Illness (CDST) |
| Scientific title | Improving Collaborative Decision Making in Veterans with Serious Mental Illness |
| Countries of recruitment | USA |
| Health condition(s) or problem(s) studied | Schizophrenia; Schizoaffective Disorder; Delusional Disorder;  Major Affective Disorder with Psychotic Features |
| Intervention(s) | Experimental: Collaborative Decision Skills Training |
|  | Control: Leveling Up |
| Key inclusion and exclusion criteria | Inclusion: currently receives services at local PRRC; has SMI diagnosis per EMR; agree to have subset of appointments audio-recorded |
|  | Exclusion: have primary substance use/organic neurological disorder; are determined by PRRC staff/study staff to be at significant risk of symptom exacerbation or risk of violence too high to manage in study setting |
| Study type | Interventional |
|  | Allocation: randomized intervention model. Parallel assignment masking: single (outcomes assessor) |
| Date of first enrollment | August 2022 |
| Target sample size | 72 |
| Recruitment status | Recruiting |
| Primary outcome(s) | Shared Decision-Making Coding System |
| Key secondary outcomes | Personal recovery, empowerment, and treatment engagement |
