# Supplementary material for: Enhancing patient-clinician collaboration during treatment decision-making: study protocol for a community-engaged, mixed method hybrid type 1 trial of collaborative decision skills training (CDST) for veterans with psychosis
Source: Trials. 2024 Jun 6;25:363. doi: 10.1186/s13063-024-08127-4 (PMC11155075; doi:10.1186/s13063-024-08127-4)
Supplement: Supplementary file 3 — Supplementary Material 3. [file 13063_2024_8127_MOESM3_ESM.pdf]

Decisional Capacity Assessment Questions
IRB: H190127
CDST

Participant ID #:_____________ Date: _____________ Total Score: _____________

1. What is the purpose of the study that was just described to you?

   Score = 2 for:
   - **Compare CDST to active control (Goal-Focused Supportive Contact group)**
   - **Help Veterans engage in assertive decision-making practices with their treatment team**
   - **Asses different treatment components between the CDST and active control groups**

   Score:

2. What makes you want to consider participating in this study?

   Score = 2 for:
   - **Feel empowered**
   - **Share my experiences**
   - **Help others**
   - **Improve mental health care for Veterans/Veterans with SMI**

   Score:

3. Do you have to be in this study if you do not want to participate?

   Score = 2 for:
   - **No**

   Score:

4. If you withdraw from this study, will you still be able to receive regular treatment at the VA?

   Score = 2 for:
   - **Yes**

   Score:

5. If you participate in this study, what activities would you do?

   Score = 2 for:
   - **Attend ten hour-long group sessions held over ten weeks**
   - **Complete a set of assessments, including three individual interviews that will be audio recorded**
   - **Complete at-home practice if participating in CDST**

Score:

6. What are the potential risks that people may experience if they participate in this study?

   Score = 2 for:
   - **Feeling uncomfortable, stressed, or having other negative emotions due to discussing negative experiences in group therapies**
   - **Short-term anxiety or discomfort from trying to change patient-provider dynamics**
   - **We do not expect any significant risks.**

   Score:

7. Will the interviews be audio recorded?

   Score = 2 for:
   - **Yes**

   Score:

8. If you have questions or concerns about this study, where can you find information to contact someone about this?

   Score = 2 for:
   - **In the IRB form**
   - **Ask Emily Treichler**
   - **Ask a study staff member**

   Score:
